# Comparative analysis of furosemide and torsemide efficacy in 24 hours of acute heart failure admission

**DOI:** 10.3389/fphar.2025.1643077

**Published:** 2025-07-31

**Authors:** Małgorzata Małek-Elikowska, Andrzej Szyszka, Julita Fedorowicz, Rafał Dankowski, Cyntia Szymańska, Artur Baszko

**Affiliations:** 2nd Department of Cardiology, Poznan University of Medical Sciences, Poznań, Poland

**Keywords:** furosemide, torsemide, acute heart failure, urinary sodium, B-lines, loop diuretics

## Abstract

**Background:**

Intravenous (IV) furosemide and torsemide represent a cornerstone of guideline-directed medical therapy for acute heart failure (AHF). However, the evidence regarding the superiority of each agent remains controversial.

**Methods:**

The prospective, open-label, comparative study included 51 adult patients hospitalized due to AHF. Torsemide was administered to 25 patients (49%), and furosemide to 26 patients (51%). The primary endpoint was the change in urinary spot sodium level at 24 h, used to assess diuretic efficacy. Secondary outcomes included lung ultrasound (LUS) B-lines, clinical status evaluation based on the Borg scale, Killip-Kimball classification, and daily urine output.

**Results:**

After 24 h of treatment, urinary sodium levels increased by an estimated marginal mean (EMM) of 21.84 mmol/L in the furosemide group and 0.97 mmol/L in the torsemide group (p = 0.173). The number of B-lines over 24 h decreased, with an EMM of 28.31 in the furosemide group, and 30.12 in the torsemide group (p = 0.779). The severity of dyspnea, measured by the Borg scale, decreased over 24 h with an EMM of 3.58 points in the furosemide group, and 3.62 points in the torsemide group (p = 0.891). Pulmonary congestion, measured by the Killip-Kimball classification, minimized with an EMM of 0.42 points in the furosemide group, and 0.47 points in the torsemide group (p = 0.770). Daily urine output after 24 h of treatment reached an EMM of 3,559.67 mL in the furosemide group, and 2,734.89 mL in the torsemide group (p = 0.068).

**Conclusion:**

Both furosemide and torsemide demonstrated comparable efficacy in the initial treatment of AHF, as assessed by laboratory, ultrasound, and clinical parameters.

## 1 Introduction

Furosemide and torsemide are the primary treatment strategies for AHF to eliminate congestion. Both medications are loop diuretics that promote renal sodium and water excretion with a rapid onset of action, helping to achieve a euvolemic state. As loop diuretics, they share the same mechanism of action. However, torsemide has a higher degree of protein binding and longer half-life, resulting in more stable natriuresis and urine output compered to furosemide. Moreover, torsemide demonstrates benefits beyond diuresis, including the inhibition of the renin-angiotensin-aldosterone system (RAAS) and sympathetic nervous activity. Because of its anti-aldosterone effect, torsemide carries a lower risk of hypokalemia. Furthermore, torsemide-treated patients showed decreased serum concentrations of the C-terminal propeptide of procollagen type I, a biochemical marker of myocardial fibrosis. Torsemide also has a neutral metabolic action, meaning it does not affect bicarbonate and lipid metabolism (McDonagh et al., 2021; Erratum, 2020; Diez et al., 2009; Lopez et al., 2004; Cuthbert et al., 2024; Felker et al., 2020). Nevertheless, the superiority of one diuretic over the other in managing AHF or chronic HF (CHF) remains unproven. A few head-to-head comparisons of loop diuretic drugs in CHF suggest a lower rate of hospitalization and cardiac mortality in torsemide treatment, along with improvements in fatigue, symptoms, and quality of life (Mullens et al., 2019; Cosín and Díez, 2002; Balsam et al., 2019; Mentz et al., 2016; Murray et al., 2001; Müller et al., 2003; Abraham et al., 2020). In contrast, other studies have shown no differences between the medications regarding hospitalization rates and 1-year all-cause mortality reduction (Mentz et al., 2016; Yao et al., 2018). To date, there have only been a limited number of studies comparing IV furosemide with torsemide in the treatment of AHF (Stroobandt et al., 1982; Paterna et al., 2011).

Diuresis and natriuresis, which occur after diuretic administration, are key indicators of diuretic response. Urine output or changes in body weight are used to assess diuresis, while measurement of sodium in a spot urine sample helps to assess natriuresis (Mullens et al., 2019). In the PUSH-AHF (Pragmatic Urinary Sodium-based Algorithm in Acute Heart Failure) and ENACT-HF (Efficacy of a Standardized Diuretic Protocol in Acute Heart Failure) studies, patients with AHF requiring treatment with IV loop diuretics were randomly assigned to natriuresis-guided therapy or standard of care. Even though no significant differences were observed between the two arms regarding all-cause mortality or first HF rehospitalization, the authors concluded that natriuresis-guided loop diuretic treatment was safe and improved natriuresis and diuresis without impacting long-term clinical outcomes (Ter Maaten et al., 2022; Dauw et al., 2024; Biegus et al., 2024). A good diuretic response within the first 24 h of AHF treatment is associated with better outcomes. Conversely, patients with poor diuretic response have worse prognosis (Cuthbert et al., 2024; Cuthbert and Clark, 2024; Felker et al., 2020; Mullens et al., 2019; Cosín and Díez, 2002). A decrease in sodium excretion during loop diuretic treatment is indicative of diuretic resistance, which refers to insufficient diuresis and natriuresis despite the administration of an adequate dose of diuretics (Paterna et al., 2011; Ter Maaten et al., 2022). The primary mechanism of resistance is intense sodium reabsorption in the distal part of the nephron, driven by high urine sodium concentration and hypochloremic alkalosis (Cox and Testani, 2020; Mullens et al., 2009; Felker et al., 2011). In the ROSE-HF (Renal Optimisation Strategies Evaluation in Acute Heart Failure) trial evaluating diuretic resistance, a lower level of urinary sodium was associated with higher 6-month mortality, unless water balance was achieved (Hodson et al., 2019). Urinary sodium levels below 50–70 mmol/L also correlate with an increased risk of renal failure and late complications (Mullens et al., 2009; Felker et al., 2011). To compare the efficacy of IV furosemide and torsemide, we assessed changes in spot urinary sodium levels during the initial treatment of AHF. Imaging modalities and clinical assessment tools play a complementary role in evaluating the efficacy of diuretic therapy. LUS has emerged as a reliable, non-invasive method to monitor pulmonary congestion by quantifying B-lines, which are reverberation artifacts representing interstitial edema. B-lines are useful for the diagnosis, monitoring, and prognostic assessment of patients with HF (Čelutkienė et al., 2020; Platz et al., 2017). A reduction in the number of B-lines over time reflects effective decongestion and has improved outcomes in HF patients (He et al., 2022). Clinical assessment tools, such as the Borg scale and the Killip-Kimball classification, offer practical insights into the severity of dyspnea and the degree of hemodynamic compromise, respectively. These tools, in conjunction with urine output, enable a multidimensional assessment of treatment response.

## 2 Methods

### 2.1 Study design and setting

This prospective, open-label, two-arm study included 51 patients aged 30–94 years who were hospitalized for AHF at the 2^nd^ Department of Cardiology, Poznan University of Medical Sciences, Poland, between February 2023 and April 2024. Patients were enrolled upon urgent hospital admission due to one of the clinical presentations of AHF: acute decompensated heart failure, acute pulmonary oedema, or isolated right ventricular failure. The diagnosis of AHF was based on clinical criteria outlined in the 2021 European Society of Cardiology Guidelines for the diagnosis and treatment of acute and chronic heart failure (McDonagh et al., 2021; Erratum, 2020). Patients were consecutively allocated to either the furosemide group or the torsemide group. Those who had not received oral loop diuretics were administered either 40 mg of IV furosemide or 20 mg of IV torsemide, while patients already on oral loop diuretics received a doubled oral dose (Supplementary Figure S1).

### 2.2 Measurement of urinary sodium and dose adjustment

Spot urinary sodium level was measured 2 h following drug administration and after 24 h. The dose of the diuretic was adjusted according to the level of urine sodium concentration. At concentrations below 70 mEq/L, a double dose was administered and repeated every 2–12 h until the dose reached 500 mg of furosemide or 100 mg of torsemide. At concentrations over 70 mEq/L, the same dose was repeated every 12 h (Supplementary Figure S1).

### 2.3 Assessment of congestion and clinical status

LUS was performed on admission and after 24 h. Quantification of congestion in LUS was based on the combined number of B-lines in the 28-zones protocol and intercostal spaces with the pleural effusion multiplied by 10. B-lines were counted between 0–10 in each area. Clinical status was evaluated using the modified Borg Dyspnoea Scale (0–10 points) and the Killip-Kimball classification (1-4 points) at admission and after 24 h. On admission, measurements included N-terminal pro-B-type natriuretic peptide (NT-proBNP), estimated glomerular filtration rate (eGFR) calculated using the Modification of Diet in Renal Disease study (MDRD) equation, electrocardiogram (ECG), and transthoracic echocardiography. Additionally, a 24-h urine collection was conducted. The local bioethics committee has approved this study, and written informed consent has been obtained from each patient (Supplementary Figure S1).

### 2.4 Statistical analysis

Analyses were conducted using the R Statistical language (version 4.3.1; R Core Team, 2023) on Windows 10 pro 64 bit (build 19,045). In the present analysis, the threshold for statistical significance was set at an alpha level of 0.05. The normality of numerical variables was evaluated using the Shapiro-Wilk test. Variables that did not adhere to a normal distribution were described by the median (Mdn) and interquartile range, specifically detailing the first (Q1) and third (Q3) quartiles. For categorical variables, the frequencies (n) and proportions of each category were detailed. For the comparison of two independent groups exhibiting non-normally distributed numerical variables, the Wilcoxon rank sum test was employed. The association between two categorical variables was assessed using either the Pearson chi-square test or Fisher’s exact test, determined by the expected frequencies within the contingency tables. The estimation of target effects was conducted through contrast analysis, examining the differences in estimated marginal means for furosemide and torsemide across both the entire cohort and specified subgroups.

## 3 Results

### 3.1 Baseline characteristics

The study cohort comprised data from 51 adult patients aged between 30–94 years, displaying a balanced gender distribution with 25 females (49.02%) and 26 males (50.98%). The age distribution had a median age of 72 years. Torsemide was administered among 25 patients (49%), whereas 26 (51%) received furosemide. The body mass index (BMI) of the cohort had a median value of 27.80 kg/m^2^. The demographic profiles of patients on torsemide and furosemide are broadly comparable, with no statistically significant differences observed regarding sex and age. However, a remarkable difference was observed in BMI between the two groups. Patients treated with torsemide had a higher median BMI (28.60 kg/m^2^) compared to those treated with furosemide (25.40 kg/m^2^), and this difference reached statistical significance (p = 0.013). The clinical profile characteristics of both groups demonstrated no significant differences regarding rates of diabetes mellitus, atrial fibrillation (AF), and hypertension. A notable difference was observed in the prevalence of chronic obstructive pulmonary disease (COPD): 24% in the torsemide group versus 61.54% in the furosemide group (p = 0.007). Kidney function, assessed by eGFR, blood pressure measurements, median ejection fraction (EF), and NT-proBNP levels showed no significant differences. However, the distribution within EF categories differed; the furosemide group had a higher percentage of patients with HF with preserved EF (HFpEF), while the torsemide group had more patients with HF with mildly reduced EF (HFmEF) (Table 1).

**TABLE 1:** Baseline characteristics of the sample.

Characteristic	Overall cohort n = 51	Torsemide-group, n = 25^a^	Furosemide-group, n = 26^a^	*p-value*
Sex				0.328
Female	25 (49.02%)	14 (56.00%)	11 (42.31%)	
Male	26 (50.98%)	11 (44.00%)	15 (57.69%)	
Age, years	72.00 (66.00, 81.00)^b^	70.00 (65.00, 82.00)^b^	73.50 (67.50, 79.75)^b^	0.571^d^
BMI, kg/m^2^	27.80 (24.10, 31.15)^b^	28.60 (27.00, 34.30)^b^	25.40 (23.60, 29.23)^b^	0.013^d^
Comorbidities, cardiac functions, and clinical conditions				
Diabetes mellitus	26 (50.98%)	13 (52.00%)	13 (50.00%)	0.886
COPD	22 (43.14%)	6 (24.00%)	16 (61.54%)	0.007
Atrial fibrillation	25 (49.02%)	14 (56.00%)	11 (42.31%)	0.328
Hypertension	26 (50.98%)	14 (56.00%)	12 (46.15%)	0.482
Ejection fraction, %	43.00 (27.50, 50.00)^b^	42.00 (30.00, 46.00)^b^	45.00 (24.00, 53.75)^b^	0.748^d^
Classification of heart failure				0.176^e^
HFmEF	10 (19.61%)	7 (28.00%)	3 (11.54%)	
HFpEF	18 (35.29%)	6 (24.00%)	12 (46.15%)	
HFrEF	23 (45.10%)	12 (48.00%)	11 (42.31%)	
Hemodynamic and laboratory parameters				
NT-proBNP, pg/mL	7.328.00 (3,996.50, 12.359.50)^b^	5.504.00 (3,953.00,12.233.00)^b^	8.523.00 (4,885.50,13,980.75)^b^	0.231^d^
eGFR, mL/min/1.73m^2^	60.00 (39.75, 60.00)^b^	60.00 (35.50, 60.00)^b^	54.90 (41.55, 60.00)^b^	0.887^d^
SBP, mmHg	131.00 (116.00, 160.00)^b^	140.00 (117.00, 165.00)^b^	127.50 (112.00, 149.25)^b^	0.193^d^
DBP, mmHg	80.00 (70.00, 88.50)^b^	83.00 (74.00, 91.00)^b^	78.50 (70.00, 86.50)^b^	0.439^d^

^a^ *n* (%).

^b^ Median (first quartile Q1, and third quartile Q3).

^c^ Pearson’s chi-square test.

^d^ Wilcoxon rank sum test.

^e^ Fisher’s exact test.

Abbreviations: BMI, body mass index; COPD, chronic obstructive pulmonary disease; HFpEF, heart failure with preserved ejection fraction; HFmEF, heart failure with mildly reduced ejection fraction; HFrEF, heart failure with reduced ejection fraction; NT-proBNP, N- terminal pro-B-type natriuretic peptide; eGFR, estimated glomerular filtration rate; SBP, systolic blood pressure; DBP, diastolic blood pressure.

### 3.2 Spot urinary sodium level changes over a 24-h period of furosemide and torsemide treatment, adjusted for sex and age

**FIGURE 1:**
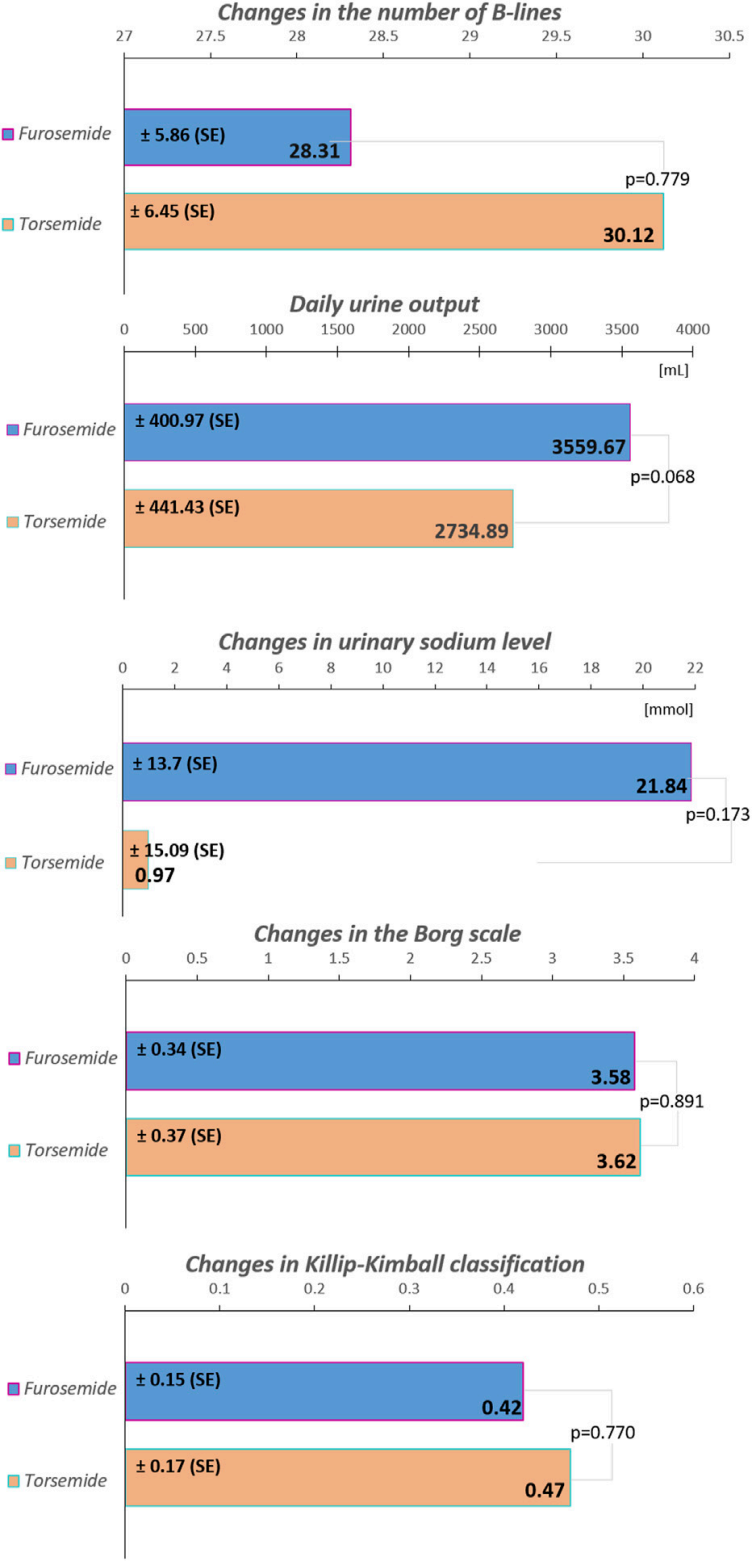
The increase in spot urinary sodium levels, the decrease in the number of B-lines, as well as in the Borg scale and Killip-Kimball classification, along with the volume of daily urine output in the total cohort during the first 24 h of treatment. The values are presented as estimated marginal means ± SE, standard error. The modified Borg Dyspnea Scale assesses ratings of perceived shortness of breath from 0 (nothing at all) to 10 (maximum); the Killip-Kimball classification assesses the severity of heart failure: 1) no clinical signs, 2) mild pulmonary edema, 3) severe pulmonary edema, 4) cardiogenic shock.

In the overall cohort without stratification, the results indicate a moderate increase in spot urinary sodium levels for patients treated with furosemide compared to those treated with torsemide, with a mean difference of 20.87 mmol/L. However, the statistical significance of this finding is weak (p = 0.173) (Figure 1). When dissecting the data into subgroups based on the presence of specific conditions, the variations in sodium levels exhibited a consistent pattern, with furosemide frequently showing higher sodium excretion compared to torsemide, although without reaching statistical significance. For instance, in the subgroup with AF, furosemide led to a 4.45 mmol/L increase in sodium levels for 24 h, while torsemide led to a 3.41 mmol/L decrease. Similarly, the subgroup without AF showed a considerable increase in sodium level with furosemide (17.96 mmol/L), and a decrease with torsemide (15.44 mmol/L), again without statistical significance (p = 0.706 and p = 0.134) (Table 2).

**TABLE 2:** Spot urinary sodium level changes over a 24-h period of furosemide and torsemide treatment, adjusted for sex and age.

Characteristic	Furosemide		Torsemide		p-value
	*EMM*	*SE*	*EMM*	*SE*	
Overall cohort	21.84	13.70	0.97	15.09	0.173
Without atrial fibrillation	17.96	12.90	−15.44	18.10	0.134
Atrial fibrillation	4.45	16.10	−3.41	14.10	0.706
Without COPD	16.04	16.70	−11.97	12.30	0.179
COPD	9.32	13.50	2.29	21.40	0.782
BMI <30 kg/m^2^	12.86	11.70	−8.96	14.10	0.239
BMI ≥30 kg/m^2^	6.52	23.60	−9.08	17.40	0.596
eGFR <60 mL/min/1.73m^2^	10.65	14.20	−0.42	16.20	0.612
eGFR ≥60 mL/min/1.73m^2^	13.80	14.80	−17.09	15.10	0.152
Without HFpEF	7.37	14.20	−16.06	12.00	0.219
HFpEF	17.30	14.70	5.63	20.80	0.641
Without HFmEF	14.23	10.30	−13.50	11.70	0.078
HFmEF	−2.99	29.60	6.73	18.60	0.784
Without HFrEF	13.27	13.50	4.42	14.40	0.651
HFrEF	11.12	15.90	−24.19	15.50	0.116

Abbreviations: EMM, estimated marginal mean; SE, standard error; COPD, chronic obstructive pulmonary disease; BMI, body mass index; eGFR, estimated glomerular filtration rate; HFpEF, heart failure with preserved ejection fraction; HFmEF, heart failure with mildly reduced ejection fraction; HFrEF, heart failure with reduced ejection fraction.

### 3.3 The effect of medication type on a change in B-lines, severity of dyspnea on the Borg scale, and Killip-Kimball classification of pulmonary congestion over 24 hours, adjusted for sex and age

**FIGURE 2:**
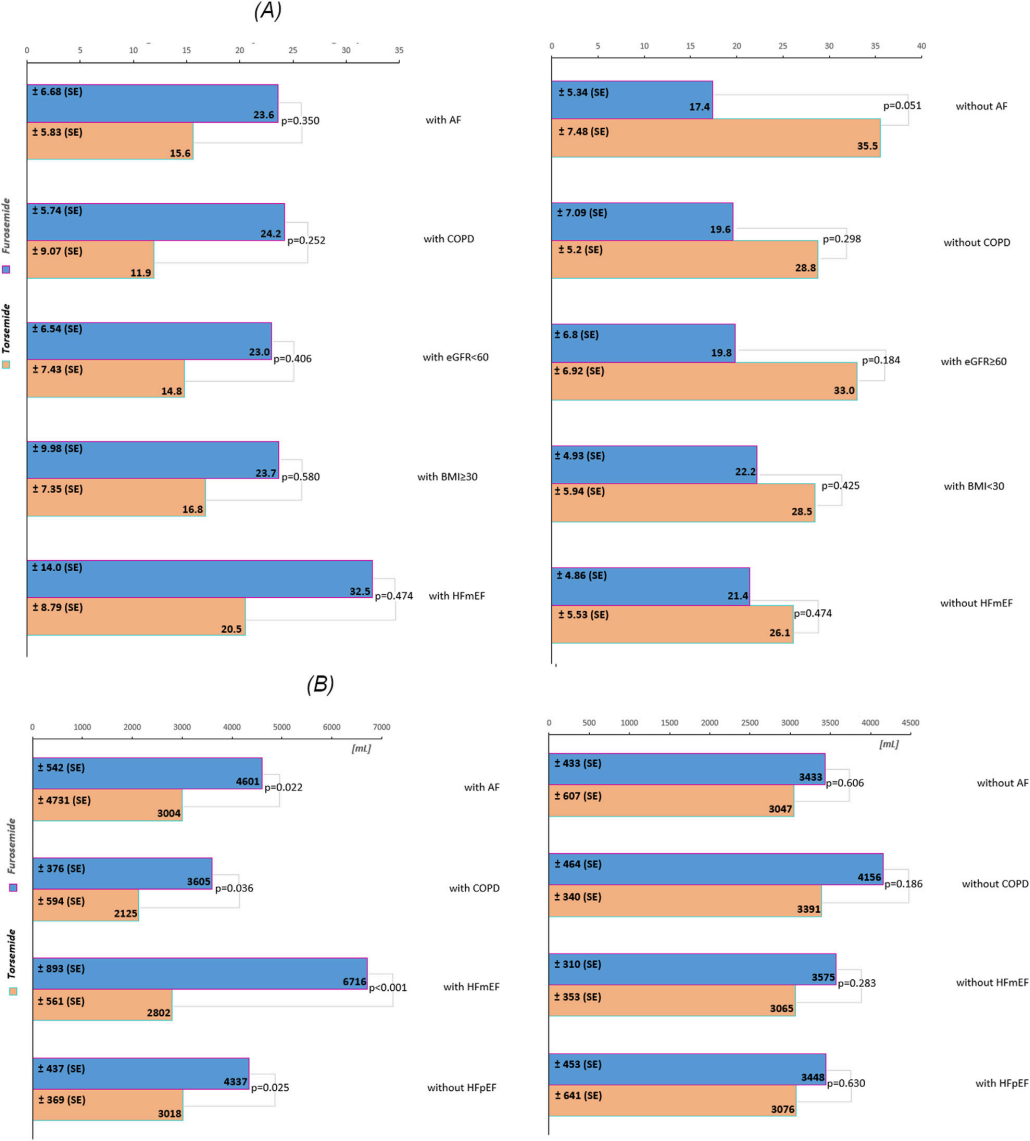
**(A)** The decrease in the number of B-lines across subgroups during the first 24 h of treatment. **(B)** The volume of daily urine output across subgroups during the first 24 h of treatment. The values are presented as estimated marginal means. Abbreviations: SE, standard error; AF, atrial fibrillation; COPD, chronic obstructive pulmonary disease; eGFR, estimated glomerular filtration rate (mL/min/1.73 m^2^); BMI, body mass index (kg/m^2^), HFpEF, heart failure with preserved ejection fraction; HFmEF, heart failure with mildly reduced ejection fraction.

In the overall cohort without stratification, the difference in B-lines reduction between furosemide and torsemide was minimal (1.82), with a non-significant p-value (p = 0.779) (Figure 1). In the subgroup without AF, furosemide showed a significantly less reduction in B-lines compared to torsemide (18.04), almost reaching statistical significance (p = 0.051). Similar non-significant trends were observed in subgroups without COPD, and without HFmEF, as well as in subgroups with eGFR ≥60 mL/min/1.73 m^2^, and with BMI<30 kg/m^2^ (Figure 2). Conversely, in patients with AF, COPD, eGFR <60 mL/min/1.73 m^2^, BMI ≥30 kg/m^2^, and HFmEF furosemide seems to perform better, reducing B-lines more than torsemide, but this difference was not statistically significant (Figure 2A). The change in the Borg scale between furosemide and torsemide is almost negligible (0.05) across the overall cohort with a non-significant p-value (p = 0.891). Exploring further into subgroup analyses reveals subtle variations, yet non-reaching statistical significance (Figure 1; Supplementary Table S1). The minimal difference (0.02) in the change of the Killip-Kimball classification between the two medications in the overall cohort, with a non-significant p-value (p = 0.770) suggests that neither furosemide nor torsemide has a distinct advantage over the other when considering the broader population of patients with AHF (Figure 1). When delving into the subgroup analyses, the results remain consistently non-significant, indicating a similar pattern across various patient demographics and clinical conditions (Supplementary Table S1).

### 3.4 Comparative efficacy of medications on daily urine output, adjusted for sex and age

In the overall cohort without stratification, torsemide resulted in a lower daily urine output 2,734.89 mL compared to furosemide 3,559.67 mL, with a decrease of 824.78 mL (Figure 1). Notably, in subgroups defined by the presence of AF and COPD, the differences were more pronounced and statistically significant. In patients with AF, furosemide increased urine output by 1,597.00 mL compared to torsemide, with a p = 0.022, indicating a significant improvement. Similarly, in the COPD subgroup, the increase with furosemide is 1,480 mL, and the difference was also statistically significant (p = 0.036). The stratification by HF type revealed further distinctions. In the HFmEF subgroup, furosemide showed a higher increase in urine output of 3,914 mL compared to torsemide, with a highly significant p-value (p < 0.001). This indicates a robust effect of furosemide in this subgroup. Likewise, in the subgroup without HFpEF, urine output significantly increased with furosemide (1,319 mL, p = 0.025). However, in other subgroups such as those defined by the absence of AF, COPD, HFmEF, and with HFpEF, the differences between the medications were not statistically significant (Figure 2B).

## 4 Discussion

Numerous clinical trials have compared the effects of torsemide and furosemide in CHF; however, relatively few have focused on AHF. The TORIC (Torsemide in Congestive HF) trial found that patients on torsemide had lower total and cardiac mortality with RRR (Relative Risk Reduction) of 51.5% and 59.7%, respectively, than those on furosemide, and presented better clinical improvement assessed by NYHA classification (Cosín and Díez, 2002). The TORNADO (Torsemide in Hemodynamic and Neurohormonal Stress and Cardiac Remodelling in HF) study demonstrated improvement of NYHA class in the torsemide group among patients with CHF (Balsam et al., 2019). Conversely, the TRANSFORM (Torsemide Comparison with Furosemide for Management of HF), a trial comparing furosemide to torsemide in CHF, suggested no difference in all-cause hospitalization or all-cause mortality (Mentz et al., 2023). Similarly, a *post hoc* analysis of the ASCEND-HF (Acute Study of Clinical Effectiveness of Nesiritide in Decompensated Heart Failure) trial compering patients discharged on torsemide versus furosemide showed no significant difference in 30-day mortality or HF rehospitalization (Mentz et al., 2016). Clasen et al. (1988) found that the diuretics had similar effects on fluid and sodium excretion, but torsemide led to less RAAS activation. In turn, analyzing features of IV loop diuretics, Paterna et al. (2011), concluded that high-doses of IV torsemide are equivalent to high-doses of IV furosemide in the treatment of refractory CHF. They included 87% of patients (n = 3,620) on furosemide and 13% (n = 557) on torsemide. Torsemide was associated with similar outcomes on unadjusted analysis and nominally lower events on adjusted analysis (30-day mortality/HF hospitalization). The other study, which included twelve patients with AHF, revealed that the effects of both drugs on urinary electrolyte and water excretion were similar (Stroobandt et al., 1982). These data suggest the potential additional value of torsemide in the treatment of CHF, possibly due to its ability to inhibit myocardial fibrosis, RAAS, and sympathetic nervous activity. However, this therapeutic advantage was not evident during the initial phase of AHF management in our study, consistent with the observations reported by Paterna and Stroobandt. Considering that furosemide performs better in sodium excretion and urine output in our study, it may point to its ability to overcome diuretic resistance in AHF, but clinical effect, that is reduction of B-lines, changes in the Borg scale, and the Killip-Kimball classification, do not vary compared to torsemide. This might imply that while furosemide could potentially be more effective at promoting sodium and urine excretion, the lack of universal statistical significance calls for cautious interpretation. The 2021 European Society of Cardiology (ESC) Guidelines for the diagnosis and treatment of AHF and CHF emphasize early and effective decongestion, recommending the prompt use of IV loop diuretics to alleviate symptoms and reduce pulmonary and systemic congestion (McDonagh et al., 2021; Erratum, 2020). Both furosemide and torsemide are recommended, although furosemide remains the most widely studied and utilized agent. Our study aligns with these guideline-directed principles, showing that both IV furosemide and torsemide achieved decongestive effects across multiple parameters, including urinary sodium excretion, reduction in B-lines, and clinical congestion scoring without significant differences. This reflects the ESC’s emphasis on monitoring the response to diuretics, such as changes in dyspnea and natriuresis, to guide therapy titration. Notably, the ESC also endorses the use of natriuresis-guided diuretic strategies to improve short-term decongestion, as seen in the ENACT-HF trial, which is mirrored in our study design through repeated urinary sodium assessments to optimize dosing (Dauw et al., 2024). The findings from our study suggest that while both agents fit within guideline-recommended decongestion strategies, the choice between furosemide and torsemide may be individualized based on patient phenotype, including comorbid conditions like AF and COPD, which in our study were associated with better urine output with furosemide. This personalized approach is consistent with the ESC’s call for tailoring therapy to individual patient characteristics and treatment response.

## 5 Limitations

This study has several noteworthy limitations. First, the open-label design may have introduced performance and detection bias, as awareness of treatment allocation could have influenced both clinical decision-making and outcome assessment. Second, based on the primary endpoint that is change in urinary spot sodium levels at 24 h, we performed a sample size estimation using an assumed standard deviation of 25 mmol/L and a mean difference of 21 mmol/L between groups, as observed in our preliminary results. We estimated that approximately 23 patients per group would be required to achieve 80% power at a p-value of 0.05. However, in a *post hoc* power analysis using observed medians and interquartile ranges, we estimated standard deviations of 56.3 mmol/L and 31.85 mmol/L for the torsemide and furosemide groups, respectively. This yielded a moderate effect size (Cohen’s d ≈ 0.54), but a reduced power of approximately 48%. This suggests that the study may have been underpowered to detect a statistically significant difference, despite a clinically meaningful trend (p = 0.097). Third, baseline imbalances were observed, particularly in the prevalence of COPD (24% vs. 61.5%, p = 0.007) and BMI (28.6 kg/m^2^ vs. 25.5 kg/m^2^, p = 0.013) between the torsemide and furosemide groups. However, multivariate analysis of these parameters did not reveal any differences in treatment outcomes between the two drugs. Fourth, this was a single-center study, which may limit the external validity and generalizability of the findings. Finally, the study focused exclusively on short-term clinical outcomes, without evaluating the impact on HF rehospitalization or mortality. Future investigations should aim to address the limitations of the present study by including larger patient populations, implementing blinding, and extending the observation period beyond 24 h. A longer evaluation window may better elucidate any delayed or sustained effects of torsemide that were not apparent during 24 h of treatment.

## 6 Conclusion

Given these results, the choice between furosemide and torsemide should be guided by clinical context and individualized patient characteristics, as the two drugs may not be equally effective in all patients. While overall differences did not consistently reach statistical significance, observed trends and subgroup variations suggest that furosemide and torsemide may exert differential effects on fluid reduction. These findings are preliminary and require validation, but they highlight the potential importance of a personalized approach to diuretic therapy in AHF and underscore the need for further research to clarify the comparative effectiveness of these agents.

## Data Availability

The original contributions presented in the study are included in the article/Supplementary Material, further inquiries can be directed to the corresponding author.

## Supplementary material

SUPPLEMENTARY FIGURE S1 Flowchart of the study. Abbreviations: AHF, acute heart failure; LUS, lung ultrasound; ECG, electrocardiogram; NT-proBNP, N- terminal pro-B-type natriuretic peptide; eGFR, estimated glomerular filtration rate; UNa, urine sodium. The modified Borg Dyspnea Scale assesses ratings of perceived shortness of breath from 0 (nothing at all) to 10 (maximum); the Killip-Kimball classification assesses the severity of heart failure: 1) no clinical signs, 2) mild pulmonary edema, 3) severe pulmonary edema, 4) cardiogenic shock.
