# Supplementary figures and images for: Comparative analysis of furosemide and torsemide efficacy in 24 hours of acute heart failure admission
Source: Front Pharmacol. 2025 Jul 31;16:1643077. doi: 10.3389/fphar.2025.1643077 (PMC12350330; doi:10.3389/fphar.2025.1643077)

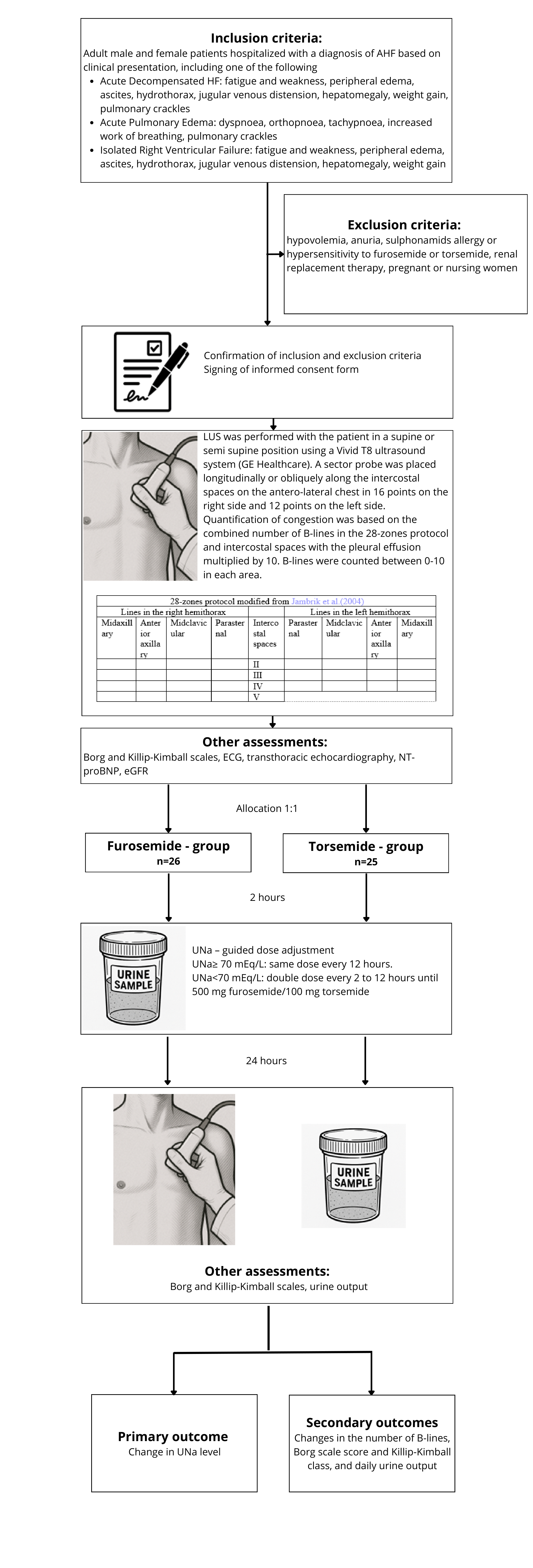

Supplement: Supplementary file 1 [file Image1.tiff]
