# Supplementary material for: Comparative analysis of furosemide and torsemide efficacy in 24 hours of acute heart failure admission
Source: Front Pharmacol. 2025 Jul 31;16:1643077. doi: 10.3389/fphar.2025.1643077 (PMC12350330; doi:10.3389/fphar.2025.1643077)
Supplement: Supplementary file 2 [file Table1.docx]

Table S1. Effect of medication type on changes in dyspnea severity (Borg scale) and pulmonary congestion (Killip-Kimball classification) over 24 hours, adjusted for age and sex

| Characteristic | Changes in the Borg scale | | | | | Changes in the Killip-Kimball classification | | | | |
| --- | --- | --- | --- | --- | --- | --- | --- | --- | --- | --- |
|  | Furosemide | | Torsemide | | *p-value* | Furosemide | | Torsemide | | *p-value* |
|  | EMM | SE | EMM | SE |  | EMM | SE | EMM | SE |  |
| Overall cohort | -3.58 | 0.34 | -3.62 | 0.37 | 0.891 | -0.42 | 0.15 | -0.47 | 0.17 | 0.770 |
| Without AF | -3.40 | 0.32 | -4.07 | 0.35 | 0.224 | -0.40 | 0.15 | -0.42 | 0.21 | 0.957 |
| AF | -3.54 | 0.40 | -3.12 | 0.35 | 0.420 | -0.65 | 0.19 | -0.60 | 0.16 | 0.843 |
| Without COPD | -3.01 | 0.36 | -3.67 | 0.26 | 0.142 | -0.30 | 0.18 | -0.65 | 0.13 | 0.113 |
| COPD | -3.76 | 0.29 | -2.96 | 0.46 | 0.141 | -0.58 | 0.14 | -0.15 | 0.22 | 0.102 |
| BMI < 30 kg/m^2^ | -3.29 | 0.27 | -3.41 | 0.27 | 0.781 | -0.43 | 0.13 | -0.42 | 0.15 | 0.946 |
| BMI ≥ 30 kg/m^2^ | -4.28 | 0.54 | -3.59 | 0.40 | 0.304 | -0.72 | 0.26 | -0.71 | 0.19 | 0.967 |
| eGFR<60mL/min/1.73m^2^ | -3.59 | 0.35 | -3.31 | 0.40 | 0.594 | -0.50 | 0.16 | -0.68 | 0.18 | 0.475 |
| eGFR≥60 mL/min/1.73m^2^ | -3.29 | 0.37 | -3.65 | 0.37 | 0.497 | -0.47 | 0.17 | -0.40 | 0.17 | 0.783 |
| Without HFpEF | -3.51 | 0.36 | -3.54 | 0.30 | 0.959 | -0.29 | 0.17 | -0.49 | 0.14 | 0.606 |
| HFpEF | -3.34 | 0.37 | -3.29 | 0.53 | 0.940 | -0.71 | 0.18 | -0.70 | 0.25 | 0.692 |
| Without HFmEF | -3.40 | 0.26 | -3.49 | 0.30 | 0.825 | -0.47 | 0.12 | -0.57 | 0.14 | 0.605 |
| HFmEF | -3.91 | 0.75 | -3.49 | 0.47 | 0.641 | -0.61 | 0.35 | -0.44 | 0.22 | 0.691 |
| Without HFrEF | -3.48 | 0.34 | -3.44 | 0.36 | 0.938 | -0.68 | 0.15 | -0.56 | 0.16 | 0.578 |
| HFrEF | -3.41 | 0.40 | -3.55 | 0.39 | 0.801 | -0.23 | 0.18 | -0.51 | 0.17 | 0.262 |

The modified Borg Dyspnea Scale assesses ratings of perceived shortness of breath from 0 (nothing at all) to 10 (maximum); the Killip-Kimball classification assesses the severity of heart failure:1) no clinical signs, 2) mild pulmonary edema, 3) severe pulmonary edema, 4) cardiogenic shock.

Abbreviations: EMM, estimated marginal mean; SE, standard error; AF, atrial fibrillation; COPD, chronic obstructive pulmonary disease; BMI, body mass index; eGFR, estimated glomerular filtration rate; HFpEF, heart failure with preserved ejection fraction; HFmEF, heart failure with mildly reduced ejection fraction; HFrEF, heart failure with reduced ejection fraction.
